# Gender differences in trends of bladder cancer mortality-to-incidence ratios according to health expenditure in 55 countries

**DOI:** 10.1371/journal.pone.0244510

**Published:** 2021-02-12

**Authors:** Cheng-Yu Huang, Shao-Chuan Wang, Lung Chan, Tzuo-Yi Hsieh, Wen-Wei Sung, Sung-Lang Chen

**Affiliations:** 1 Department of Urology, National Taiwan University Hospital, Taipei, Taiwan; 2 Department of Urology, Chung Shan Medical University Hospital, Taichung, Taiwan; 3 School of Medicine, Chung Shan Medical University, Taichung, Taiwan; 4 Institute of Medicine, Chung Shan Medical University, Taichung, Taiwan; National Yang-Ming University, TAIWAN

## Abstract

The association between bladder cancer mortality-to-incidence ratios (MIRs) and healthcare disparities has gender differences. However, no evidence supports gender as an issue in the association between changes in the MIR and health expenditures on bladder cancer. Changes in the MIR were defined as the difference in data from the years 2012 and 2018, which was named δMIR. Current health expenditures (CHE) and the human development index (HDI) were obtained from the World Health Organization and the Human Development Report Office. The association between variables was analyzed by Spearman’s rank correlation coefficient. In total, 55 countries were analyzed according to data quality and the exclusion of missing data. Globally, the MIR changed according to the HDI level in both genders. Among the 55 countries studied, a high HDI and CHE were significantly associated with a favorable age-standardized rate-based MIR (ASR-based MIR) in both genders and the subgroups according to gender (for both genders, MIR vs. HDI: ρ = -0.720, p < 0.001; MIR vs. CHE per capita: ρ = -0.760, p < 0.001; MIR vs. CHE as a percentage of gross domestic product (CHE/GDP): ρ = -0.663, p < 0.001). Importantly, in females only, the CHE/GDP but neither the HDI score nor the CHE per capita was significantly associated with a favorable ASR-based δMIR (ASR-based δMIR vs. CHE/GDP: ρ = 0.414, p = 0.002). In the gender subgroups, the association between the HDI and the CHE was statistically significant for females and less significant for males. In conclusion, favorable bladder ASR-based MIRs were associated with a high CHE; however, improvement of the ASR-based δMIR data was more correlated with the CHE in females. Further investigation of the gender differences via a cohort survey with detailed information of clinical-pathological characteristics, treatment strategies, and outcomes might clarify these issues and improve therapeutic and/or screening strategies for bladder cancer.

## Introduction

Bladder cancer is the 10^th^ most common cancer worldwide, with a total of 549,000 new cases and 200,000 deaths estimated in 2018 [1,2]. Although the exact cause of bladder cancer is unclear, recent research has clarified that certain risk factors may be related, including smoking, dietary choices, environmental carcinogens, and socioeconomic factors [2]. Smoking is a significant contributor to the risk of bladder cancer [3]. The Surveillance Epidemiology and End Results database from the United States suggests that Black people have a lower incidence rate but a higher mortality rate for bladder cancer, which may indicate that its prevalence is related to race [4]. Additionally, bladder cancer incidence and mortality seem to be higher in more developed countries [5]. A recent study demonstrated a rising trend for developing countries in Asia and Central and Eastern Europe [6]. The disparities of incidence and mortality in different genders are also well documented [2,5,7]. However, the trends of incidence and mortality regarding different regions, genders, and healthcare systems demand closer evaluation.

Gender differences in bladder cancer incidence are related to biologic and epidemiologic factors, such as disparate metabolism of carcinogens, hormonal factors, and exposure risk, including cigarette smoking and occupational exposure [8–11]. Moreover, females suffer from delays in diagnosis due to inefficiencies in healthcare delivery and their likelihood of being less responsive to intravesical therapy for non-muscle invasive bladder cancer [10,11]. These factors are related to disparities in healthcare and socioeconomic factors among countries. To investigate the gender differences in this issue, the mortality-to-incidence ratio (MIR), which is a novel parameter that is used to evaluate the diagnostic capacity and treatment quality of a certain disease, is employed. It acts as an estimation for survival and has been demonstrated as a potential indicator of the long-term success of cancer surveillance programs in colorectal cancer [12]. In our previous study, by using the 2012 GLOBOCAN database, MIRs were notably higher in less developed regions, and they had a significant association with their World Health Organization (WHO) ranking and total expenditure on health/gross domestic product (GDP) percentage [5].

Nonetheless, the previous study relied on cross-sectional data because the GLOBOCAN database only registered the mortality and incidence rates from 2012. The status of the diagnosis, treatment, and survival outcomes for bladder cancer have varied in different regions and countries over the years. However, the GLOBOCAN 2018 data (published in September 2018) can give us a more comprehensive understanding of the current trend for bladder cancer incidence and mortality. δMIR, an innovative parameter that represents data from the years 2012 and 2018, was therefore developed for further research. By comparing the MIRs from 2012 and 2018, we hope to gain a better understanding of the screening process and treatment quality for bladder cancer in different countries and regions.

## Materials and methods

Cancer epidemiological data were obtained from the GLOBOCAN project (http://gco.iarc.fr/), which is maintained by the International Agency for Research on Cancer, WHO. It is a public access database that provides contemporary estimates of cancer epidemiology for 185 countries. The exclusion criteria for country selection was based on the data quality report of GLOBOCAN (N = 121) and missing data (N = 3). Outliers of the MIR (N = 6) were also excluded. In total, 55 countries were included in the final analysis. The MIR, including the age-standardized rate (ASR) used in this survey, was obtained from the 2018 GLOBOCAN database. The human development index (HDI) score was obtained from the United Nations Development Program, Human Development Report Office (http://hdr.undp.org/en, HDI of 2017, downloaded on 07/05/2019). The data on health expenditures include current health expenditures (CHE) per capita and the CHE to the GDP in percentage (CHE/GDP), which were obtained from the World Health Statistics (https://www.who.int/gho/publications/world_health_statistics/en/, analysis of 2018, downloaded on 07/05/2019). The MIR is defined as the ratio of the crude rate (CR) of mortality and the CR of incidence, as previously described [5,12–14]. δMIR is defined as the difference between the MIR in 2012 and 2018 [δMIR = MIR (in 2012)—MIR (in 2018)] [15]. Associations between the MIR, δMIR, and other factors among various countries were estimated by Spearman’s rank correlation coefficient using SPSS statistical software version 15.0 (SPSS, Inc., Chicago, IL). P values of < 0.05 were considered statistically significant. A scatterplot was generated via Microsoft Excel.

## Results

### The HDI is related to incidence, mortality, and MIR

The CRs of incidence, mortality, and MIR in males, females, and both genders for bladder cancer are summarized in Table 1. When we classified regions according to their HDI ranking, very high HDI regions had a higher CR of incidence and mortality (male: 33.0 and 7.7, respectively; female: 8.8 and 2.3, respectively; both genders: 20.8 and 5.0, respectively) but the lowest MIR (male: 2.23; female: 0.26; both genders: 0.24). Low HDI regions had a lower CR of incidence and mortality (male: 1.4 and 0.84, respectively; female: 0.93 and 0.63, respectively; both genders: 1.2 and 0.73, respectively) but the highest MIR (male: 0.60; female: 0.68; both genders: 0.61).

**Table 1:** Summary of the crude rates and mortality-to-incidence ratio of bladder cancer according to the continents.

	Incidence, CR			Mortality, CR			MIR		
Region	All genders	Male	Female	All genders	Male	Female	All genders	Male	Female
Human development index									
Very high HDI	20.8	33.0	8.8	5.0	7.7	2.3	0.24	0.23	0.26
High HDI	5.7	8.8	2.5	2.2	3.3	1.0	0.39	0.38	0.40
Medium HDI	1.9	2.9	0.91	0.99	1.5	0.46	0.52	0.52	0.51
Low HDI	1.2	1.4	0.93	0.73	0.84	0.63	0.61	0.60	0.68
Continents									
Africa	2.2	3.0	1.3	1.2	1.6	0.8	0.55	0.53	0.61
Asia	4.0	6.2	1.7	1.5	2.3	0.7	0.38	0.37	0.41
Europe	23.6	38.4	9.6	6.5	10.5	2.7	0.28	0.27	0.28
Latin America and the Caribbean	3.9	5.6	2.3	1.4	2.0	0.8	0.36	0.36	0.37
North America	22.1	34.3	9.9	3.9	5.8	2.0	0.18	0.17	0.20
Oceania	7.5	11.8	3.1	3.0	4.5	1.5	0.40	0.38	0.48

data from Global Cancer Observatory, International Agency for Research on Cancer.

### Incidence, mortality, and MIR by continent

By continent (Table 1), Europe and North America had the highest CR of incidence (Europe: male: 38.4; female: 9.6; both genders: 23.6; North America: male: 34.3, female: 9.9; both genders: 22.1), while Africa had the lowest (male: 3.0; female: 1.3; both genders: 2.2). Three regions with a lower CR of mortality were Africa, Asia, and Latin America and the Caribbean (Africa: male: 1.6; female: 0.8; both genders: 1.2; Asia: male: 2.3; female: 0.7; both genders: 1.5; Latin America and the Caribbean: male: 2.0; female: 0.8; both genders: 1.4). Europe had the highest CR of mortality (male: 10.5; female: 2.7; both genders: 6.5). Africa had the highest MIR (male: 0.53; female: 0.61; both genders: 0.55), and North America had the lowest MIR (male: 0.17; female: 0.20; both genders: 0.18).

### Incidence, mortality, and MIRs of bladder cancer as well as the HDI and CHE by country

Table 2 summarizes the HDI, CHE, incidence, mortality, and ASR-based MIR in bladder cancer of the 55 selected countries. The 4 countries with a CR of incidence greater than 35 were Denmark (40.2), Germany (38.5), Italy (39.1), and the Netherlands (35.7). The countries with a crude mortality rate higher than 10.0 were Croatia (10.3) and Latvia (10.8).

**Table 2:** Summary of human development index, current health expenditure, cancer incidence, cancer mortality, and ASR-based mortality-to-incidence ratio in bladder cancer of selected countries (n = 55).

	Human Development Index^1^		Current Health Expenditure^2^		Incidence^3^			Mortality^3^			ASR-based MIR		
Country	Score	Rank	Per Capita	% of GDP	ASR	CR	Cum. Risk	ASR	CR	Cum. Risk	2012	2018	δMIR
Argentina	0.825	47	998	6.8	4.9	7.0	0.63	1.8	2.8	0.22	0.37	0.37	0.00
Australia	0.939	3	4934	9.4	5.1	10.6	0.63	1.7	4.0	0.19	0.26	0.33	-0.07
Belarus	0.808	53	352	6.1	7.4	13.9	0.97	1.4	2.8	0.17	0.32	0.19	0.13
Belgium	0.916	17	4228	10.5	15.3	33.8	1.95	2.6	6.8	0.30	0.18	0.17	0.01
Brazil	0.759	79	780	8.9	4.6	5.7	0.57	1.4	1.8	0.16	0.40	0.30	0.10
Bulgaria	0.813	51	572	8.2	11.7	25.8	1.50	3.4	9.0	0.44	0.29	0.29	0.00
Canada	0.926	12	4508	10.4	9.8	21.4	1.24	1.9	4.7	0.22	0.22	0.19	0.03
Chile	0.843	44	1102	8.1	4.0	6.3	0.50	1.6	2.7	0.19	0.44	0.40	0.04
Colombia	0.747	90	374	6.2	2.6	3.0	0.32	0.8	1.0	0.09	0.38	0.31	0.07
Costa Rica	0.794	63	929	8.1	2.7	3.7	0.33	1.0	1.4	0.10	0.41	0.35	0.06
Croatia	0.831	46	852	7.4	13.1	29.6	1.65	3.6	10.3	0.41	0.28	0.27	0.01
Cuba	0.777	73	826	10.9	6.1	12.0	0.79	2.3	5.0	0.29	0.43	0.38	0.05
Czechia	0.888	27	1284	7.3	11.1	25.4	1.45	2.3	6.3	0.28	0.25	0.21	0.04
Denmark	0.929	11	5497	10.3	17.0	40.2	2.20	2.7	7.3	0.30	0.27	0.16	0.11
Ecuador	0.752	86	530	8.5	2.0	2.2	0.24	0.7	0.8	0.07	0.41	0.34	0.07
Egypt	0.696	115	157	4.2	11.4	9.0	1.48	5.7	4.5	0.70	0.50	0.50	0.00
Estonia	0.871	30	1112	6.5	8.4	19.3	1.09	2.1	6.0	0.25	0.40	0.25	0.15
Fiji	0.741	92	175	3.6	2.3	2.3	0.29	0.9	1.0	0.13	0.52	0.41	0.11
Finland	0.920	15	4005	9.4	7.3	18.7	0.93	1.0	3.2	0.12	0.20	0.14	0.06
France	0.901	24	4026	11.1	8.7	20.5	1.10	2.8	7.3	0.32	0.34	0.32	0.02
Germany	0.936	5	4592	11.2	14.7	38.5	1.84	2.3	7.6	0.25	0.15	0.16	-0.01
Iceland	0.935	6	4375	8.6	12.8	23.0	1.71	1.1	2.4	0.19	0.22	0.09	0.13
Ireland	0.938	4	4757	7.8	9.6	17.5	1.21	2.0	4.4	0.21	0.25	0.21	0.04
Israel	0.903	22	2756	7.4	11.1	15.6	1.42	2.2	3.7	0.25	0.17	0.20	-0.03
Italy	0.880	28	2700	9.0	15.1	39.1	1.93	2.2	7.7	0.25	0.23	0.15	0.08
Jamaica	0.732	97	294	5.9	2.6	3.3	0.33	1.2	1.7	0.16	0.33	0.46	-0.13
Japan	0.909	19	3733	10.9	8.1	24.2	1.01	1.1	4.5	0.13	0.25	0.14	0.11
Kuwait	0.803	56	1169	4.0	4.7	2.7	0.54	2.0	1.0	0.21	0.38	0.43	-0.05
Latvia	0.847	41	784	5.8	11.4	26.0	1.52	3.8	10.8	0.47	0.39	0.33	0.06
Lithuania	0.858	35	923	6.5	7.8	18.1	0.99	2.3	6.4	0.27	0.34	0.29	0.05
Luxembourg	0.904	21	6236	6.0	7.8	15.7	0.98	2.1	4.5	0.26	0.22	0.27	-0.05
Malaysia	0.802	57	386	4.0	2.5	2.4	0.31	1.1	1.1	0.13	0.30	0.44	-0.14
Mauritius	0.790	65	506	5.5	3.0	4.9	0.38	1.2	2.0	0.12	0.42	0.40	0.02
Netherlands	0.931	10	4746	10.7	15.4	35.7	1.98	2.5	6.6	0.28	0.37	0.16	0.21
Norway	0.953	1	7464	10.0	12.2	25.4	1.55	1.5	4.0	0.16	0.19	0.12	0.07
Oman	0.821	48	636	3.8	4.8	2.6	0.52	1.6	0.8	0.18	0.52	0.33	0.19
Philippines	0.699	113	127	4.4	1.7	1.4	0.21	0.8	0.6	0.09	0.47	0.47	0.00
Poland	0.865	33	797	6.3	12.9	26.5	1.69	4.3	9.8	0.54	0.36	0.33	0.03
Portugal	0.847	41	1722	9.0	7.6	19.4	0.95	2.4	7.5	0.27	0.23	0.32	-0.09
Russian Federation	0.816	49	524	5.6	5.9	10.8	0.78	2.0	4.0	0.26	0.44	0.34	0.10
Serbia	0.787	67	491	9.4	14.9	28.9	1.92	3.9	9.1	0.49	0.30	0.26	0.04
Singapore	0.932	9	2280	4.3	4.0	7.8	0.49	1.3	2.7	0.15	0.21	0.33	-0.12
Slovakia	0.855	38	1108	6.9	10.2	19.9	1.33	3.5	7.7	0.43	0.24	0.34	-0.10
Slovenia	0.896	25	1772	8.5	9.8	22.8	1.25	2.2	6.6	0.25	0.35	0.22	0.13
South Africa	0.699	113	471	8.2	3.2	2.8	0.38	1.5	1.3	0.17	0.41	0.47	-0.06
South Korea	0.903	22	2013	7.4	3.6	7.5	0.45	1.0	2.5	0.11	0.27	0.28	-0.01
Spain	0.891	26	2354	9.2	14.7	34.1	1.91	2.8	8.0	0.33	0.29	0.19	0.10
Sweden	0.933	7	5600	11.0	10.5	25.2	1.35	1.8	5.1	0.20	0.23	0.17	0.06
Switzerland	0.944	2	9818	12.1	11.9	26.8	1.52	2.0	5.3	0.23	0.18	0.17	0.01
Thailand	0.755	83	217	3.8	2.5	4.2	0.32	1.3	2.2	0.16	0.48	0.52	-0.04
Trinidad and Tobago	0.784	69	1146	6.0	3.4	4.9	0.44	1.6	2.4	0.20	0.36	0.47	-0.11
Ukraine	0.751	88	125	6.1	6.5	12.7	0.85	2.5	5.4	0.33	0.42	0.38	0.04
United Kingdom	0.922	14	4356	9.9	6.2	14.9	0.77	2.1	5.9	0.23	0.50	0.34	0.16
United States of America	0.924	13	9536	16.8	11.2	22.1	1.42	1.7	3.8	0.20	0.21	0.15	0.06
Uruguay	0.804	55	1281	9.2	8.7	15.1	1.13	2.5	5.0	0.31	0.38	0.29	0.09

^1^data from Human Development Reports, United Nations Development Programme.

^2^data from World Health Statistics 2018, World Health Organization.

^3^data from Global Cancer Observatory, International Agency for Research on Cancer.

Regarding the ASR-based MIR in 2018, Egypt (0.50) was the only country with ASR-based MIRs ≥ 0.50, while only one country had a MIR < 0.10 (Iceland 0.09). ASR-based δMIR was defined as the ASR-based MIR in 2012 minus the ASR-based MIR in 2018 to help us compare the changes in MIR between those two years. The two countries with the highest ASR-based δMIR were Oman (0.19) and the Netherlands (0.21). Those with an ASR-based δMIR ≤ -0.10 were Slovakia (-0.10), Trinidad and Tobago (-0.11), Singapore (-0.12), Jamaica (-0.13), and Malaysia (-0.14). Tables 3 and 4 analyze the gender differences in the association of MIR and other parameters, data of incidence, mortality, and MIR of bladder cancer divided by gender.

**Table 3:** Summary of cancer incidence, cancer mortality, and ASR-based mortality-to-incidence ratio in bladder cancer of male in selected countries (n = 55).

	Incidence^1^			Mortality^1^			Mortality-to-incidence Ratio		
Country	ASR	CR	Cum. Risk	ASR	CR	Cum. Risk	2012	2018	δMIR
Argentina	8.9	11.3	1.12	3.2	4.4	0.39	0.38	0.36	0.02
Australia	8.4	16.9	1.02	2.7	6.0	0.29	0.26	0.32	-0.06
Belarus	15.2	23.4	1.97	3.1	5.0	0.39	0.35	0.20	0.15
Belgium	25.6	53.9	3.22	4.3	10.7	0.49	0.18	0.17	0.01
Brazil	6.9	7.9	0.86	2.2	2.6	0.25	0.39	0.32	0.07
Bulgaria	20.2	40.8	2.58	6.0	13.7	0.76	0.32	0.30	0.02
Canada	15.7	33.3	1.97	3.1	7.2	0.35	0.22	0.20	0.02
Chile	6.5	9.2	0.80	2.5	3.7	0.29	0.45	0.38	0.07
Colombia	4.1	4.4	0.52	1.2	1.4	0.14	0.35	0.29	0.06
Costa Rica	4.0	5.2	0.49	1.4	2.0	0.15	0.39	0.35	0.04
Croatia	22.0	45.9	2.76	6.2	15.4	0.70	0.32	0.28	0.04
Cuba	9.7	18.3	1.25	3.5	7.1	0.43	0.41	0.36	0.05
Czechia	18.4	38.4	2.37	3.7	9.0	0.44	0.26	0.20	0.06
Denmark	27.3	62.5	3.47	4.0	10.5	0.45	0.26	0.15	0.11
Ecuador	2.9	2.9	0.34	0.9	1.0	0.10	0.35	0.31	0.04
Egypt	18.6	13.6	2.37	9.6	6.8	1.13	0.51	0.52	-0.01
Estonia	15.5	30.1	2.00	4.1	9.3	0.47	0.45	0.26	0.19
Fiji	3.5	3.5	0.44	1.5	1.5	0.20	0.56	0.43	0.13
Finland	12.7	30.4	1.58	1.8	5.0	0.20	0.20	0.14	0.06
France	15.6	34.9	1.97	4.9	12.1	0.56	0.33	0.31	0.02
Germany	24.3	61.2	3.01	3.6	11.3	0.39	0.14	0.15	-0.01
Iceland	20.9	37.1	2.67	2.0	4.2	0.38	0.27	0.10	0.17
Ireland	14.3	25.6	1.79	2.8	5.9	0.29	0.25	0.20	0.05
Israel	20.3	26.7	2.58	4.0	6.1	0.45	0.17	0.20	-0.03
Italy	25.7	64.1	3.28	3.9	12.6	0.44	0.24	0.15	0.09
Jamaica	3.7	4.6	0.48	1.9	2.4	0.25	0.36	0.51	-0.15
Japan	13.7	39.4	1.71	1.8	6.6	0.21	0.24	0.13	0.11
Kuwait	6.7	4.0	0.78	2.6	1.4	0.28	0.35	0.39	-0.04
Latvia	19.8	37.9	2.52	8.7	18.9	1.05	0.47	0.44	0.03
Lithuania	15.0	28.9	1.87	4.9	10.6	0.58	0.43	0.33	0.10
Luxembourg	13.6	25.6	1.68	3.8	7.2	0.49	0.22	0.28	-0.06
Malaysia	3.9	3.6	0.48	1.7	1.6	0.20	0.31	0.44	-0.13
Mauritius	4.7	6.9	0.64	1.8	2.7	0.21	0.48	0.38	0.10
Netherlands	23.8	54.3	3.02	3.8	9.8	0.41	0.35	0.16	0.19
Norway	19.1	38.5	2.41	2.4	6.0	0.24	0.19	0.13	0.06
Oman	7.0	3.4	0.83	2.2	0.9	0.27	0.51	0.31	0.20
Philippines	2.7	1.9	0.33	1.3	0.9	0.13	0.48	0.48	0.00
Poland	22.4	41.6	2.90	8.1	16.0	0.99	0.40	0.36	0.04
Portugal	13.5	32.0	1.69	4.4	12.5	0.50	0.22	0.33	-0.11
Russian Federation	11.9	18.0	1.56	4.4	6.9	0.56	0.48	0.37	0.11
Serbia	24.9	45.5	3.21	7.0	14.5	0.86	0.32	0.28	0.04
Singapore	6.7	12.3	0.79	2.0	3.8	0.24	0.21	0.30	-0.09
Slovakia	17.3	29.8	2.22	6.8	12.6	0.81	0.25	0.39	-0.14
Slovenia	15.6	34.2	1.97	3.6	9.7	0.38	0.35	0.23	0.12
South Africa	5.8	4.1	0.68	2.7	1.8	0.29	0.40	0.47	-0.07
South Korea	6.6	12.4	0.80	1.9	3.8	0.19	0.27	0.29	-0.02
Spain	25.5	56.6	3.32	5.2	13.6	0.60	0.29	0.20	0.09
Sweden	16.4	38.4	2.10	2.7	7.3	0.30	0.23	0.16	0.07
Switzerland	19.2	42.1	2.42	3.2	8.1	0.35	0.17	0.17	0.00
Thailand	4.4	6.8	0.55	2.3	3.6	0.28	0.49	0.52	-0.03
Trinidad and Tobago	6.2	8.3	0.79	3.0	4.2	0.37	0.37	0.48	-0.11
Ukraine	13.2	21.9	1.74	5.5	9.7	0.73	0.46	0.42	0.04
United Kingdom	9.5	22.1	1.17	3.0	8.2	0.32	0.47	0.32	0.15
United States of America	18.3	34.4	2.30	2.7	5.7	0.31	0.20	0.15	0.05
Uruguay	16.8	26.1	2.15	4.8	8.2	0.58	0.41	0.29	0.12

^1^data from Global Cancer Observatory, International Agency for Research on Cancer.

**Table 4:** Summary of cancer incidence, cancer mortality, and ASR-based mortality-to-incidence ratio in bladder cancer of female in selected countries (n = 55).

	Incidence^1^			Mortality^1^			Mortality-to-incidence Ratio		
Country	ASR	CR	Cum. Risk	ASR	CR	Cum. Risk	2012	2018	δMIR
Argentina	1.8	2.8	0.22	0.7	1.3	0.10	0.33	0.39	-0.06
Australia	2.0	4.3	0.25	0.8	2.0	0.10	0.31	0.40	-0.09
Belarus	2.4	5.4	0.31	0.3	0.8	0.00	0.26	0.13	0.13
Belgium	6.0	13.7	0.75	1.0	3.0	0.10	0.21	0.17	0.04
Brazil	2.7	3.6	0.33	0.8	1.2	0.10	0.38	0.30	0.08
Bulgaria	4.8	11.4	0.59	1.5	4.5	0.20	0.24	0.31	-0.07
Canada	4.3	9.5	0.54	0.8	2.1	0.10	0.23	0.19	0.04
Chile	2.0	3.3	0.24	0.9	1.6	0.10	0.45	0.45	0.00
Colombia	1.3	1.6	0.16	0.5	0.6	0.10	0.54	0.38	0.16
Costa Rica	1.5	2.1	0.17	0.5	0.8	0.10	0.50	0.33	0.17
Croatia	5.8	14.2	0.71	1.6	5.4	0.20	0.23	0.28	-0.05
Cuba	2.8	5.7	0.35	1.3	2.8	0.20	0.54	0.46	0.08
Czechia	4.9	12.6	0.64	1.3	3.8	0.20	0.27	0.27	0.00
Denmark	7.3	17.8	0.95	1.5	4.0	0.20	0.33	0.21	0.12
Ecuador	1.3	1.5	0.15	0.5	0.6	0.10	0.43	0.38	0.05
Egypt	5.0	4.2	0.67	2.5	2.2	0.30	0.48	0.50	-0.02
Estonia	3.6	9.6	0.45	0.9	3.0	0.10	0.36	0.25	0.11
Fiji	1.0	1.1	0.15	0.4	0.5	0.10	0.44	0.40	0.04
Finland	2.6	7.1	0.32	0.4	1.4	0.10	0.26	0.15	0.11
France	2.4	6.2	0.29	0.9	2.6	0.10	0.45	0.38	0.07
Germany	5.9	16.0	0.73	1.1	3.8	0.10	0.22	0.19	0.03
Iceland	5.0	8.5	0.74	0.2	0.6	0.00	0.10	0.04	0.06
Ireland	5.2	9.4	0.65	1.2	2.9	0.10	0.26	0.23	0.03
Israel	2.9	4.4	0.36	0.7	1.3	0.10	0.21	0.24	-0.03
Italy	5.7	14.6	0.69	0.8	2.9	0.10	0.23	0.14	0.09
Jamaica	1.5	2.1	0.19	0.7	1.0	0.10	0.32	0.47	-0.15
Japan	2.9	9.2	0.34	0.5	2.4	0.10	0.32	0.17	0.15
Kuwait	1.9	1.0	0.19	1.1	0.6	0.10	0.59	0.58	0.01
Latvia	5.8	15.7	0.84	0.9	3.8	0.10	0.23	0.16	0.07
Lithuania	3.1	8.6	0.39	0.7	2.7	0.10	0.21	0.23	-0.02
Luxembourg	2.4	5.6	0.29	0.6	1.8	0.10	0.23	0.25	-0.02
Malaysia	1.1	1.2	0.14	0.5	0.6	0.10	0.29	0.45	-0.16
Mauritius	1.6	2.8	0.17	0.6	1.3	0.00	0.33	0.38	-0.05
Netherlands	7.5	17.0	0.96	1.3	3.3	0.20	0.45	0.17	0.28
Norway	5.5	11.9	0.70	0.8	1.9	0.10	0.20	0.15	0.05
Oman	1.7	1.1	0.14	0.9	0.5	0.10	0.52	0.53	-0.01
Philippines	0.9	0.8	0.10	0.4	0.4	0.00	0.44	0.45	-0.01
Poland	5.5	12.2	0.70	1.5	3.9	0.20	0.30	0.27	0.03
Portugal	2.7	8.0	0.32	0.8	3.0	0.10	0.26	0.30	-0.04
Russian Federation	2.0	4.4	0.26	0.5	1.4	0.10	0.37	0.25	0.12
Serbia	6.4	12.8	0.79	1.5	3.9	0.20	0.29	0.23	0.06
Singapore	1.6	3.3	0.20	0.7	1.6	0.10	0.22	0.44	-0.22
Slovakia	4.9	10.5	0.62	1.2	3.0	0.10	0.28	0.24	0.04
Slovenia	4.7	11.3	0.58	1.1	3.5	0.10	0.41	0.23	0.18
South Africa	1.5	1.5	0.17	0.8	0.8	0.10	0.45	0.53	-0.08
South Korea	1.1	2.5	0.13	0.4	1.1	0.00	0.35	0.36	-0.01
Spain	5.3	12.0	0.63	0.9	2.6	0.10	0.30	0.17	0.13
Sweden	4.8	11.7	0.62	0.9	2.8	0.10	0.28	0.19	0.09
Switzerland	5.1	11.5	0.65	0.9	2.6	0.10	0.22	0.18	0.04
Thailand	1.0	1.8	0.12	0.5	0.9	0.10	0.50	0.50	0.00
Trinidad and Tobago	1.0	1.6	0.14	0.4	0.7	0.10	0.33	0.40	-0.07
Ukraine	2.0	4.6	0.26	0.6	1.7	0.10	0.28	0.30	-0.02
United Kingdom	3.3	7.9	0.40	1.3	3.5	0.10	0.61	0.39	0.22
United States of America	4.8	10.0	0.61	0.9	2.0	0.10	0.24	0.19	0.05
Uruguay	2.3	4.4	0.28	0.9	2.0	0.10	0.28	0.39	-0.11

^1^data from Global Cancer Observatory, International Agency for Research on Cancer.

### The association between the HDI, the CHE per capita, the CHE/GDP percentage, the ASR-based MIR, and δMIR

**Fig 1:**
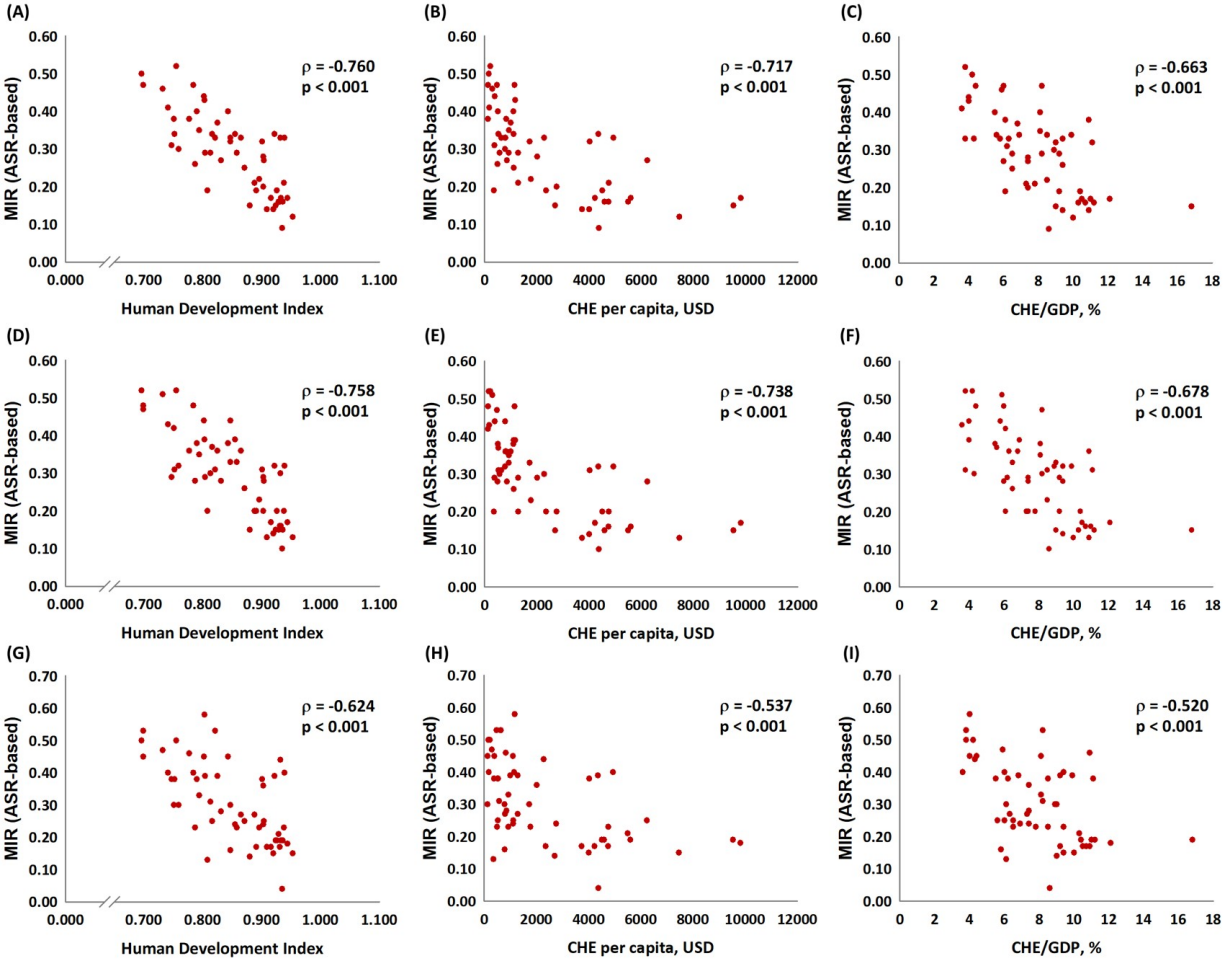
The association between the ASR-based mortality-to-incidence ratio, the human development index, the current health expenditure per capita, and current health expenditure as a percentage of gross domestic product in both genders (A to C), male patients (D to F), and female patients (G to I) with bladder cancer.

**Fig 2:**
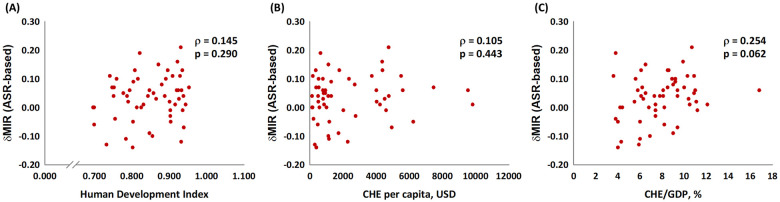
The association between (A) the human development index, (B) the current health expenditure per capita, and (C) the current health expenditure as a percentage of the gross domestic product and the ASR-based delta mortality-to-incidence ratio (δMIR, ASR-based) in bladder cancer of both genders.

**Fig 3:**
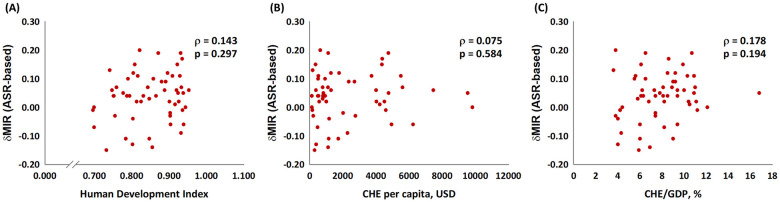
The association between (A) the human development index, (B) the current health expenditure per capita, and (C) the current health expenditure as a percentage of gross domestic product and the ASR-based delta mortality-to-incidence ratio (δMIR, ASR-based) in male bladder cancer patients.

**Fig 4:**
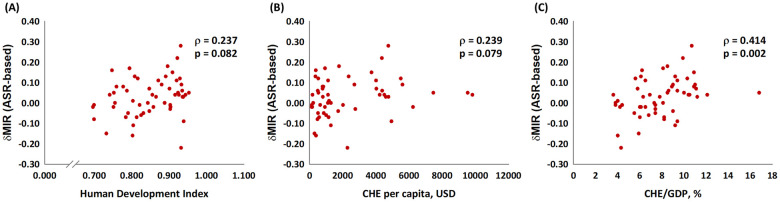
The association between (A) the human development index, (B) the current health expenditure per capita, and (C) the current health expenditure as a percentage of the gross domestic product and the ASR-based delta mortality-to-incidence ratio (δMIR, ASR-based) in female bladder cancer patients.

**Fig 5:**
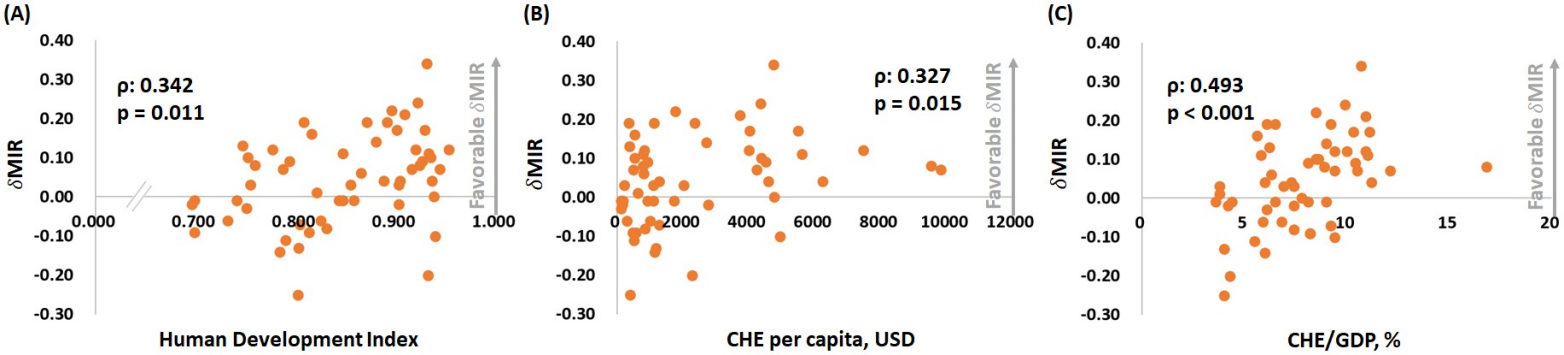
The association between (A) the human development index, (B) the current health expenditure per capita, and (C) the current health expenditure as a percentage of the gross domestic product and the CR-based delta mortality-to-incidence ratio (δMIR) in female bladder cancer patients.

Fig 1 depicts the association between the ASR-based MIR and the HDI, the CHE per capita, and the CHE/GDP percentage. As in the graphic, the ASR-based MIR showed a significant negative correlation to all three indicators in males, females, and both genders for bladder cancer. These indicators are essential for healthcare disparity evaluation. Countries with a higher HDI, a higher CHE per capita, and a higher CHE/GDP percentage tend to have a lower bladder cancer MIR. We aimed to introduce the ASR-based δMIR as an innovative parameter for the improvement of healthcare systems. Fig 2 shows a trend toward a positive correlation between the ASR-based δMIR and the CHE/GDP percentage (ρ = 0.254, p = 0.062, Fig 2C) but not for the CHE per capita and the HDI (p > 0.2, Fig 2A and 2B). As we further divide the data by gender, the ASR-based δMIR for male patients fails to demonstrate such correlations in the HDI and the CHE per capita (Fig 3A–3C), while the ASR-based δMIR for female patients significantly and positively correlates with the CHE/GDP percentage (ρ = 0.414, p = 0.002, Fig 4C) and trends toward a positive correlation of the HDI and the CHE per capita (Fig 4A and 4B). The correlation of the HDI and the CHE is statistically significant in CR-based δMIR for female patients (Fig 5A–5C).

## Discussion

To our knowledge, this is the first article to adopt the 2018 GLOBOCAN database for bladder cancer MIR research. The results showed strong correlations between the ASR-based MIR and the HDI, the CHE per capita, and the CHE/GDP percentage. Given that these parameters showed positive correlations with WHO healthcare rankings in a previous study [16], the results support the conclusion that the MIR acts as an effective evaluator of healthcare disparity. Notably, Europe and North America remain the two regions with the highest bladder cancer incidence. This may be attributed to advanced disease detection and exposure to risk factors [2]. This study was also the first to use δMIR as a parameter. The results showed a positive correlation between δMIR and the CHE/GDP. The country with the poorest δMIR was Malaysia, dropping to a stunning -0.14. The regression validated that δMIR serves as a potential evaluator for improved detection and management of bladder cancer. An association between the δMIR and true improvement in healthcare ranking with the same criteria by a future study will further prove the case.

Another interesting finding was the gender disparities in our results. The δMIR was strongly associated with the HDI and the CHE per capita for female patients; it even reached statistical significance with the CHE/GDP (Fig 5A–5C). For male patients, the δMIR failed to show any significant correlation with all three parameters (S3 Fig). The differences in bladder cancer presentation and disease progression between males and females are fascinating and have been well documented [2,5,7,17]. While male patients are three-fold more likely to develop bladder cancer, female patients often present with later-stage disease, which has been identified as an adverse prognostic factor [18]. The cause of such disparities has not yet been determined, although legitimate possibilities include smoking habits, tumor biology, occupational risk factors, and sex steroid hormones and their receptors [5]. Sex steroid hormones and their receptors might contribute to a relatively high incidence of males developing bladder cancer, but females generally present with more advanced disease with worse oncologic outcomes [19]. These results indicate the potential role of sex steroid hormones as therapeutic targets in the future. In 2016, Dobrunch et al. conducted an overview of gender and bladder cancer and stated that female patients often experience a significantly greater delay in urologic referral, and they undergo guideline-concordant imaging less frequently when encountering hematuria [11]. These findings might explain the positive correlation between the CHE/GDP and the improvement of MIR, especially in females. A better healthcare system with more funding available may augment resources to overcome these delays and offer optimal treatment for such patients. Moreover, the molecular underpinning of the endocrine and hormone pathways and the subsequent exploration potential of novel gender-specific diagnostic and management strategies would be an essential focus in studying the gender differences found with bladder cancer.

Heterogeneous age groups across nations might be a confounding factor in this survey. Given that the MIR is calculated from the ratio of mortality and incidence of a CR, the possible confounding factor of heterogeneous age groups across nations was not adjusted. Therefore, we used an ASR-based MIR to investigate gender differences in bladder cancer. The original results using CR-based analyses still showed strong correlations between the MIR and the HDI, the CHE per capita, and the CHE/GDP percentage in both genders (all p < 0.001, S1 Fig). In the analysis of CR-based δMIR of all populations and males, a significant association between the ASR-based δMIR and CHE/GDP (but not the HDI) and CHE per capita (S2 and S3 Figs) were all significant in females (Fig 5). This evidence proved the effects of heterogeneous age and gender groups across nations regarding bladder cancer.

This study has other limitations, including that the cross-sectional database of GLOBOCAN only offers a limited understanding of the disease’s trend. By comparing the data from 2012 to that of 2018, we hope to generate better insight into the tendency for bladder cancer. The innovative parameter δMIR might need further investigation to ensure its value and statistical testing for the equality of two correlation coefficients. However, its positive correlation with common healthcare evaluation indicators provides essential credit. The use of the CHE/GDP as an assessment for healthcare quality is debatable. Nonetheless, a positive correlation was found between the total expenditure on health/GDP percentage and the WHO’s rankings in our previous study [5]. A comprehensive evaluation of all healthcare systems worldwide is needed to generate a more reliable parameter.

## Conclusions

The MIR for bladder cancer serves as a legitimate indicator for healthcare system evaluation. The ASR-based δMIR is an innovative and effective criterion for the improvement of treatment and screening plans for bladder cancer. The advancement of identification and management for female patients may contribute to improvements in combatting bladder cancer.

## Supporting information

S1 Fig The association between CR-based mortality-to-incidence ratio, the human development index, the current health expenditure per capita, and current health expenditure as a percentage of gross domestic product in both genders (A to C), male patients (D to F), and female patients (G to I) with bladder cancer. (DOCX)

S2 Fig The association between (A) the human development index, (B) the current health expenditure per capita, and (C) the current health expenditure as a percentage of the gross domestic product and the CR-based delta mortality-to-incidence ratio (δMIR) in bladder cancer of both genders. (DOCX)

S3 Fig The association between (A) the human development index, (B) the current health expenditure per capita, and (C) the current health expenditure as a percentage of gross domestic product and the CR-based delta mortality-to-incidence ratio (δMIR) in male bladder cancer patients. (DOCX)
