# Supplementary material for: Gender differences in trends of bladder cancer mortality-to-incidence ratios according to health expenditure in 55 countries
Source: PLoS One. 2021 Feb 12;16(2):e0244510. doi: 10.1371/journal.pone.0244510 (PMC7880433; doi:10.1371/journal.pone.0244510)
Supplement: S1 Fig — (DOCX) [file pone.0244510.s001.docx]

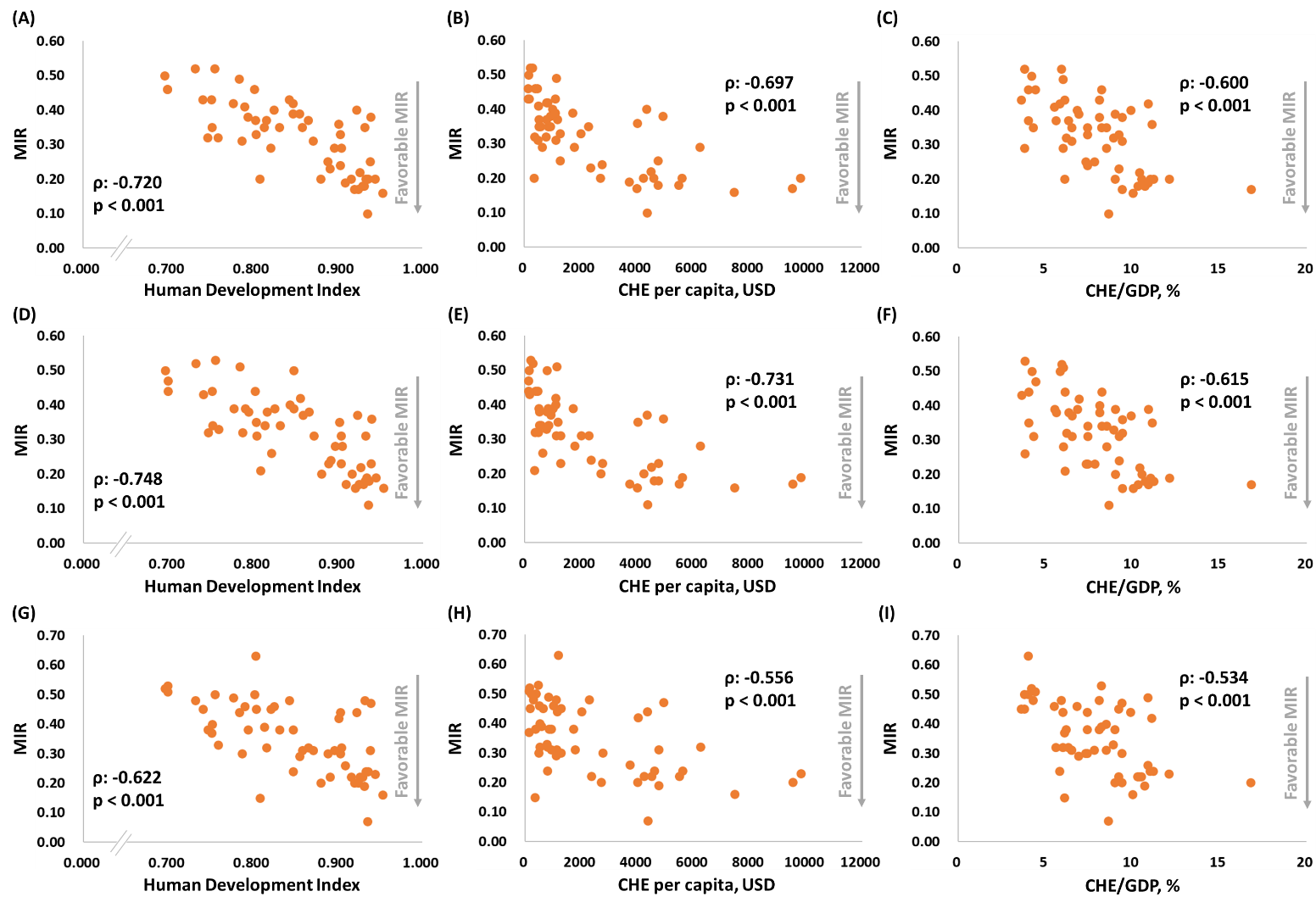


S1 Fig. The association between CR-based mortality-to-incidence ratio, the human development index, the current health expenditure per capita, and current health expenditure as a percentage of gross domestic product in both genders (A to C), male patients (D to F), and female patients (G to I) with bladder cancer.
