# Supplementary material for: Gender differences in trends of bladder cancer mortality-to-incidence ratios according to health expenditure in 55 countries
Source: PLoS One. 2021 Feb 12;16(2):e0244510. doi: 10.1371/journal.pone.0244510 (PMC7880433; doi:10.1371/journal.pone.0244510)
Supplement: S2 Fig — (DOCX) [file pone.0244510.s002.docx]

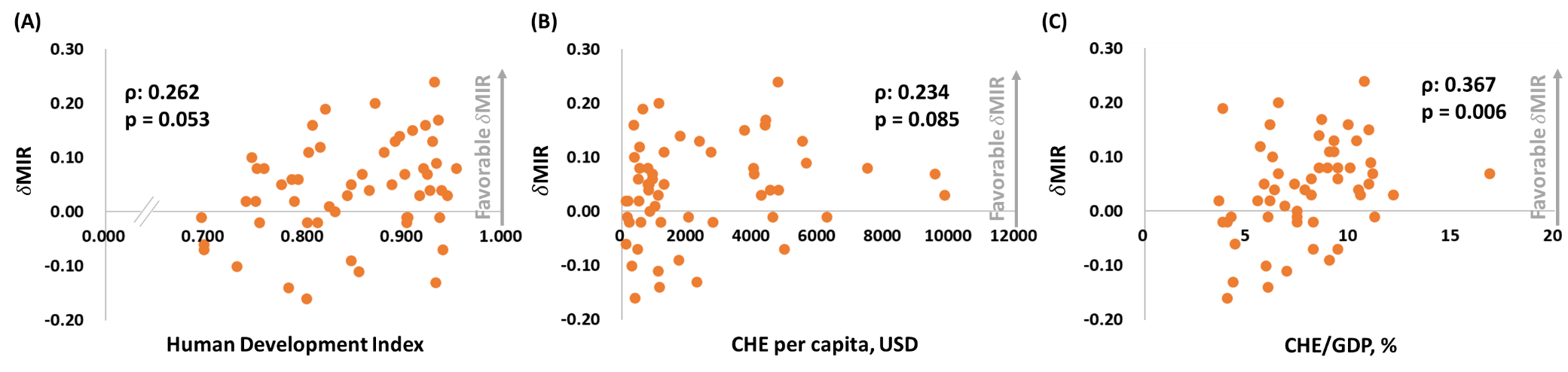


S2 Fig. The association between (A) the human development index, (B) the current health expenditure per capita, and (C) the current health expenditure as a percentage of the gross domestic product and the CR-based delta mortality-to-incidence ratio (δMIR) in bladder cancer of both genders.
